# Exploration of interprofessional collaboration for the diagnosis of infections and antibiotic prescription in nursing homes using multiple case study observational research

**DOI:** 10.1093/jacamr/dlae205

**Published:** 2025-01-13

**Authors:** Damien Gonthier, Laetitia Ricci, Marie Buzzi, Gabriel Birgand, Joëlle Kivits, Nelly Agrinier

**Affiliations:** Inserm, INSPIIRE, Université de Lorraine, Nancy F-54000, France; Centre Régional en Antibiothérapie, CHRU-Nancy, Nancy F-54000, France; Inserm, INSPIIRE, Université de Lorraine, Nancy F-54000, France; CIC, Epidémiologie Clinique, INSERM, CHRU-Nancy, Université de Lorraine, Nancy F-54000, France; Inserm, INSPIIRE, Université de Lorraine, Nancy F-54000, France; CIC, Epidémiologie Clinique, INSERM, CHRU-Nancy, Université de Lorraine, Nancy F-54000, France; Regional Center for Infection Prevention and Control, Region of Pays de la Loire, Nantes University Hospital, Nantes, France; Inserm, ECEVE, Université Paris Cité, Paris F-75010, France; Inserm, INSPIIRE, Université de Lorraine, Nancy F-54000, France; CIC, Epidémiologie Clinique, INSERM, CHRU-Nancy, Université de Lorraine, Nancy F-54000, France

## Abstract

**Background:**

Antibiotic resistance in nursing homes (NHs) is inconsistently tackled by antimicrobial stewardship programmes. The literature on individual determinants of antibiotic prescriptions (APs) in NHs is extensive. However, less is known about the structural determinants of AP in NHs.

**Objectives:**

To examine how different organizational contexts influenced interprofessional collaboration in the diagnosis and treatment of infections in NHs.

**Methods:**

We conducted multiple case study observational research through field notes and sketches from pictures of NH layouts.

**Results:**

We observed three NHs for 10 days (i.e. 82 h). We inductively identified four successive steps: (i) trigger by an assistant nurse, (ii) internal decision-making, (iii) calling on an external general practitioner (GP) and (iv) GP intervention. Diagnosis and treatment of infections involved various degrees of interprofessional collaboration within NHs, resulting in a range of actions, more or less directly involving AP by external GPs. In the case of onsite AP, external GPs barely relied on information about residents provided by NH professionals and did not provide any feedback regarding their decision, resulting in limited interprofessional collaboration. In contrast, remote AP (through phone calls) relied on interprofessional collaboration through mandatory exchanges between external GPs and NH nurses about the resident’s symptoms and signs.

**Conclusions:**

Diagnosis and treatment of infections through AP involved two distinct organization types (institutional versus private practices) and often lacked interprofessional collaboration. Future antimicrobial stewardship in NHs should consider (i) improving the connection between these two organizations and (ii) developing tools to support remote interprofessional collaboration to sustain prescription.

## Introduction

Given the progressive improvement in living conditions in recent decades, the proportion of people over 60 years of age in the world population is expected to double between 2015 and 2050,^1,2^ and dependence related to aging is expected to grow accordingly. In 2019, 7504 nursing homes (NHs) accommodated ∼585 000 residents in France. In 2016, a European cross-sectional survey estimated that the proportion of residents receiving antibiotics was 4.9% (95% CI: 4.8–5.1)^3^ and that the proportion of residents presenting at least one active infection in French NHs was 2.93% (95% CI: 2.57–3.29).^4^ Thus, there is a gap between the prevalence of infections and the prevalence of antibiotic prescribing, which results in increased antibiotic resistance. Moreover, a recent meta-analysis across 39 countries indicated that only 28.5% (95% CI: 10.3–58.0) of antibiotic prescriptions (APs) in NHs were appropriate.^5^

In France, resistance to amoxicillin/clavulanate, fluoroquinolones and ceftriaxone from *Enterobacterales* (*Escherichia coli*, *Proteus mirabilis* and *Klebsiella pneumoniae*) cultured from urine samples collected from 2014 to 2017 was 40% higher among NH residents than their community-dwelling peers.^6^ APs appear as the main modifiable factor to avoid an increase in antimicrobial resistance, supporting the need for antimicrobial stewardship (AMS) implementation in NHs.^7^

According to the WHO, AMS consists in a systematic approach aiming to educate and support healthcare professionals to follow evidence-based guidelines for prescribing and administering antibiotic treatments.^8^

AMS interventions in hospital settings are effective in increasing compliance with antibiotic policies and reducing the duration of antibiotic treatment.^9^ However, maximizing the impact of such initiatives in NHs remains challenging.^10^ The varying organizational contexts observed in NHs may hinder the proper implementation of AMS programmes tailored to hospital settings. In addition, the AMS programmes developed in hospital settings might fail to target determinants of APs that are specific to NHs. Among these determinants, some relate to residents, such as the frequency of cognitive impairment and poor or atypical symptoms of infections in elderly individuals,^11,12^ which can contribute to diagnostic uncertainties and result in subsequent inappropriate prescription of antibiotics. Other determinants of AP, pertaining to specific organizational contexts, might also be missed as a target by AMS programmes tailored to hospital settings.^13^

French NHs are primarily residential locations where social and nursing support is provided to residents [essentially by nurses and assistant nurses (ANs)]. An onsite coordinating general practitioner (GP) and a coordinating nurse manage organizational matters.^14^ However, the primary care of residents is mainly delivered by their GP from private practices that are external to NHs.^15^ Such an organization might hinder interprofessional communication. In fact, staff heavy workload and high turnover were identified in the literature to prevent building trust between nursing staff and GPs,^16^ while decisions made by GPs often rely on the clinical information provided by onsite nurses.^17^

As AP in NHs relies mainly on interprofessional collaboration,^18^ examining how infections in various NH organizations are diagnosed and treated is critical. The objective of this work was therefore to examine how various organizational contexts influence interprofessional collaboration in the diagnosis and treatment of infections.

## Materials and methods

### Study design

We performed multiple case study observational research^19^ that allows the collection of different types of data, such as observations, interviews with stakeholders or photographs.^20^ The present study is reported according to the COnsolidated criteria for REporting Qualitative research.^21^

### Sampling

We selected NHs located in the French Region Grand-Est (more than 5.5 million inhabitants in 2021). The identification of NHs was based on an up-to-date complete list of 615 NHs.

As 85% of French NHs are not integrated in a hospital and therefore do not have an in-house pharmacy,^15^ we selected NHs without a an in-house pharmacy. In the selected NHs, private GPs (resident’s GP or on-call GP) performed AP. Several criteria were considered (see Table 1) to conduct heterogeneous sampling (maximal variation).^19^ Agreement of NH directors was obtained after complete information. The director then informed the health professionals, residents and families about the study. No health professional refused to participate or reported any discomfort related to the data collection method.

**Table 1.** Description of included nursing homes

	Geographical location (number of inhabitants)^a^	Funding model	Number of residents	Number of GPs^b^	Coordination	Number of health professionals working per day-shift
**Nursing Home 1**	Semirural (2851)	Public	80	15	Medical + nurse	1 Nurse 7 ANs
**Nursing Home 2**	Urban (10 046)	Private non-profit	40	24	Medical + nurse	2 Nurses 4 ANs
**Nursing Home 3**	Rural (319)	Private for profit	24	2	Only nurse	1 Nurse 3 ANs

ANs, assistant nurses; GPs, general practitioners.

^a^Number of inhabitants of the city where the NH was located in 2020 (https://www.insee.fr/fr/statistiques).

^b^Number of private general practitioners working per NH.

### Data collection

An observation grid was designed to investigate interprofessional collaboration for the diagnosis and treatment of infections.^22^

During observations, we took field notes without any audio recording of exchanges between the observer and the stakeholders. We also sketched the facility layouts using pictures.

D.G. conducted the data collection from February 2019 to June 2019. D.G. is a male primary care M.D. with a master’s degree in public health, including training in observational methods.

### Analysis

An inductive thematic analysis was conducted based on the field notes. D.G. proposed a draft of the thematic grid after the observation of the first NH. The thematic grid was then tested and refined on the basis of field notes from another NH observation during a meeting with a PhD health sociologist. Data analysis was conducted under the supervision of a PhD, MD specializing in public health. The recruitment ended after the saturation point was reached, i.e. when sufficient data were obtained to report on all aspects of the phenomenon.^23^ The three experts involved in the analysis (D.G., J.K. and N.A.) collectively decided when the saturation point was reached. No software was used for the qualitative data analysis.

### Ethics

A declaration of compliance MR004 was made to the *Commission Nationale Informatique et Liberté* (CNIL registration number 2220997). Oral consent was obtained from all the health professionals observed. The directors gave their oral consent to sketches of the facility layouts from pictures.

## Results

The observation lasted 82 h, divided into 3 days in NH 1 and 2 and 4 days in NH 3, covering at least two different day shifts and one shift handover in each NH.

**Figure 1.**
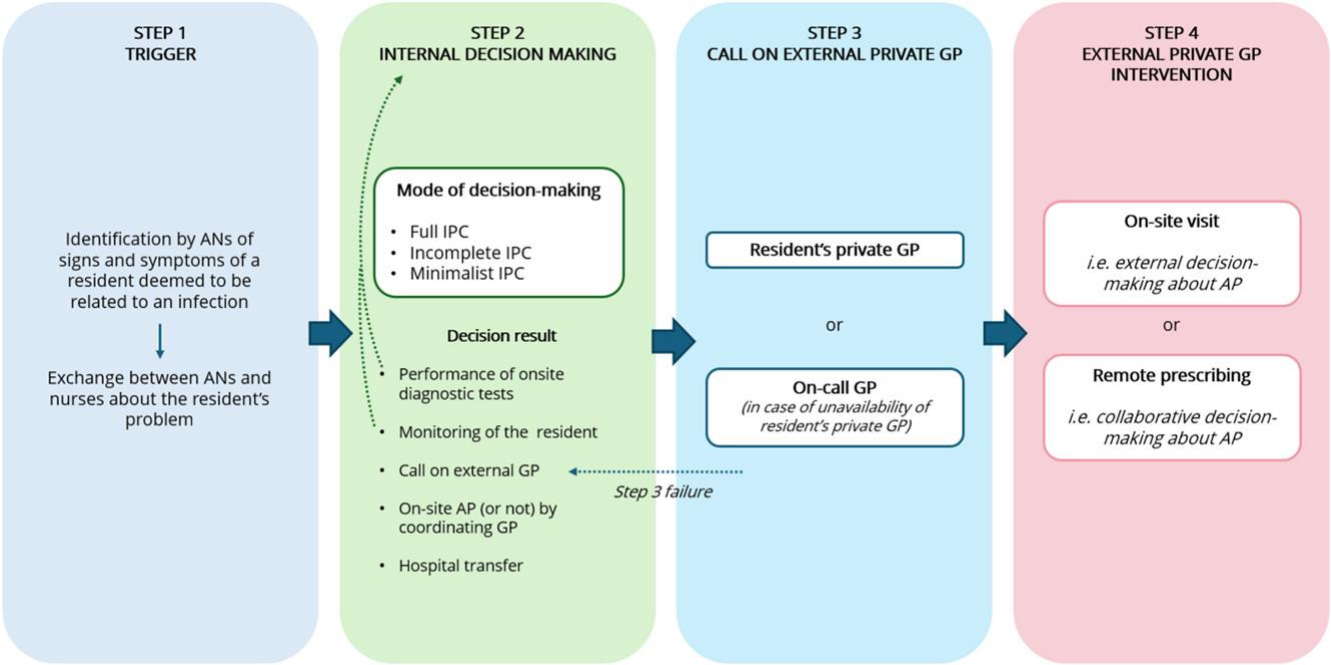
Steps in the diagnosis and treatment of infections in nursing homes. ANs, assistant nurses; AP, antibiotic prescription; IPC, interprofessional collaboration.

Four successive steps were identified in the diagnosis and treatment of infections (Figure 1).

### Step 1: trigger

The management of infections was initiated by an AN, who noticed early suggestive signs in the patient and informed the nurses. This frontline position was perceived as very important by ANs, who presented it as an expression of their will to help the resident feel better.

Observations at handover in NH 1, Day 1

ANs talk among themselves. They observed a change in behaviour and pain signs for a resident. An AN tells me: ‘I informed the nurse quickly. I felt bad for him; I knew he needed medical care fast. It’s my caregiver role’.

However, nurses’ lack of trust in AN’s clinical skills sometimes hindered this first step. This was particularly salient when nurses had to work with substitute ANs (in the absence of incumbent ANs).

Observations at the beginning of the afternoon of Day 1, NH 1

In this NH, ANs and nurses organize their handover at different times and different places every day. During the handover, an AN talks about the situation of a resident presenting with cough and marked asthenia during the visit. She indicates to me that she has informed the nurse of this problem and that she thinks the resident needs a medical visit. She does not claim to know what treatment he needs but she knows that his condition is unusual and that he presents a disease. However, the nurse has a different opinion, and she doubts the quality of the information provided by the AN: ‘The assistant nurses and us, we don’t do the same work. It’s not negative, but for them, it’s mostly admissions and discharges, what the resident is eating and whether he’s going to the bathroom’. Ultimately, the morning nurse asks the afternoon nurse to monitor the resident’s status.

Observations in the morning of Day 2, NH 3

In the nursing room, an AN informs the nurse of a resident’s increased confusion with significant aggressiveness during nursing care. She says that urine smelled and thinks that a urinary tract infection could be the cause of his confusion. The nurse shows signs of annoyance -and once the AN has left—tells me: ‘She doesn’t know this resident; it’s always the same with these substitutes, they don’t know how to deal with the residents’. Nevertheless, she decides to examine the resident, to perform a urinary test, and to call the resident’s GP in the afternoon.

### Step 2: internal decision-making

In this step, health professionals collaborated to determine the most appropriate action to take regarding an event deemed infectious.

NH health professionals could consider various options: contact the resident’s private GP, perform onsite microbiological tests, or monitor the resident’s health status until evidence of worsening, if any. This decision-making process depended mostly on organizational factors specific to the NH. In NH 1, the nurse on shift made the decision on her own. In NH 3, the nurse made the decision on her own but collected input from ANs during the afternoon nursing staff meeting. In NH 2, a meeting including all professionals—i.e. the onsite coordinating physician and the coordinating nurse, the nurses, the ANs, the psychologist and the animator—occurred daily to discuss significant events. The onsite coordinating physician and the coordinating nurse led the meeting and collected insights from each professional to make a consensual decision.

### Step 3: call on an external private GP

This step consisted of contact with the resident’s private GP or an on-call private GP.

Once the decision to contact the resident’s private GP was made, nurses often encountered difficulties (for instance, waiting on the phone or being blocked because the call was not passed on to the GP). To address these issues, the coordinating nurse of NH 2 adapted the organization practices by freeing up dedicated time during her shifts to contact GPs. Moreover, the onsite coordinating physician took care of the patient when the resident’s private GP was unavailable.

Observations after a nursing staff meeting in the afternoon of Day 3, NH 2

A urine culture was performed on a resident. The results showed the presence of bacteria, and antibiotic susceptibility testing results were available. The nurse tried to contact the resident’s private GP in the morning. The GP’s secretary had taken the message, but the referring GP did not call back or visit the resident. The onsite coordinating physician decided to prescribe an antibiotic and then called the resident’s GP directly on his private phone.

Some nurses anticipated these difficulties and directly contacted an on-call private GP when they knew from experience that the resident’s GP showed poor responsiveness.

Observations in the morning of Day 3, NH 3

When I arrive at NH 3, I notice that a resident returned from a local hospital after a one-day hospitalization to investigate a fever. The nurse explains to me that the fever had been noticed around 6 pm and that the resident’s private GP was never available at that time of day. She therefore decided, without contacting the resident’s private GP beforehand, to call the emergency department, which decided to hospitalize the resident.

### Step 4: external private GP intervention

Two factors affected the interprofessional collaboration between GPs and nurses when they came to visit a resident.

First, we noted the influence of the number of private GPs involved in visiting residents. In NHs 1 and 2, the high number of private referring GPs increased the diversity of prescription habits. In contrast, in NH 3, only two residents’ private GPs were involved in visiting the residents, and nurses could therefore adapt to them more easily.

Observations at the end of the morning on Day 2 in NH 2

Looking at a resident’s chart, a nurse understands that an antibiotic had been prescribed by the private GP based on the results of a urine culture, but she had not been informed about it. I ask the nurse about this prescription. She tells me this is usual, and she knows that she has to keep an eye on the prescription chart on the desk. She seems annoyed because it makes her work more difficult. She says, ‘How do you want to organize something with so many different general practitioners [visiting residents]?’

Observations in the afternoon on Day 1 NH 3

A nurse about the number of residents’ private GPs visiting residents: ‘There are only two residents’ private GPs working in the NH. We know them well, we have privileged contacts with them, and we know their working habits. We need them because there are few of them. We adapt to them’.

**Figure 2.**
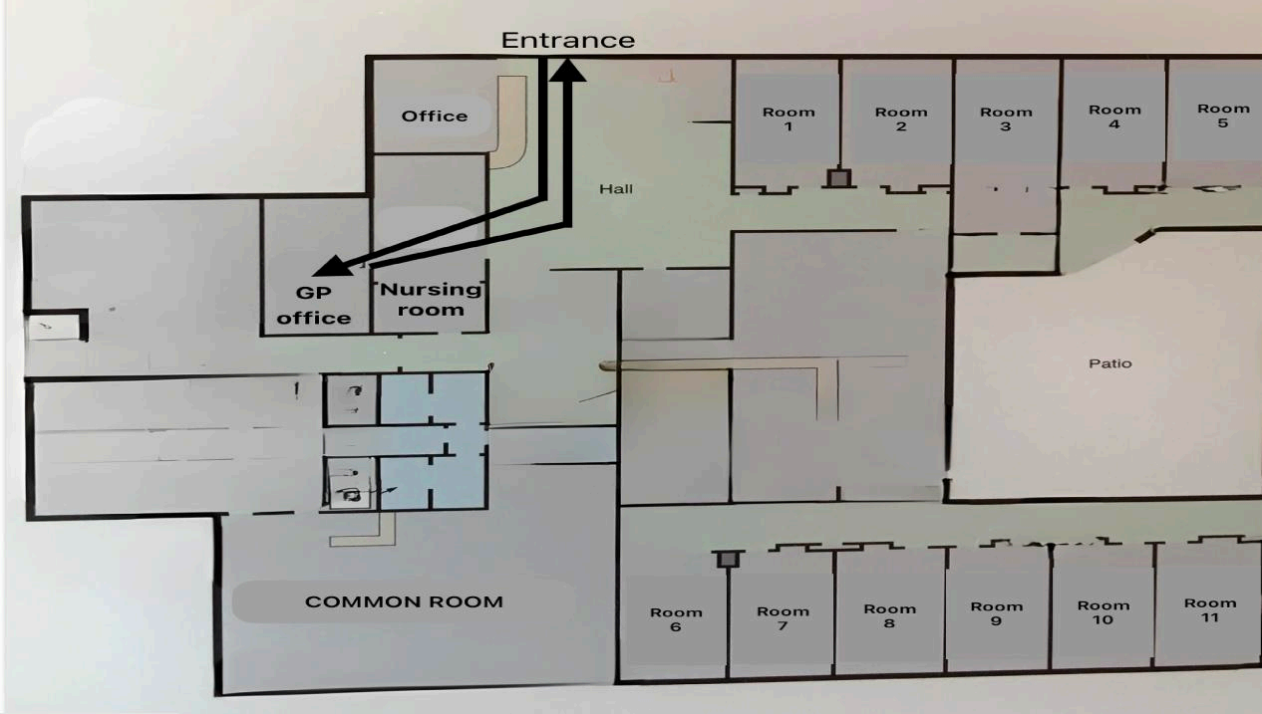
Pathway of a GP's visit to NH 3. GP, general practitioner. Photo by the first author.

**Figure 3.**
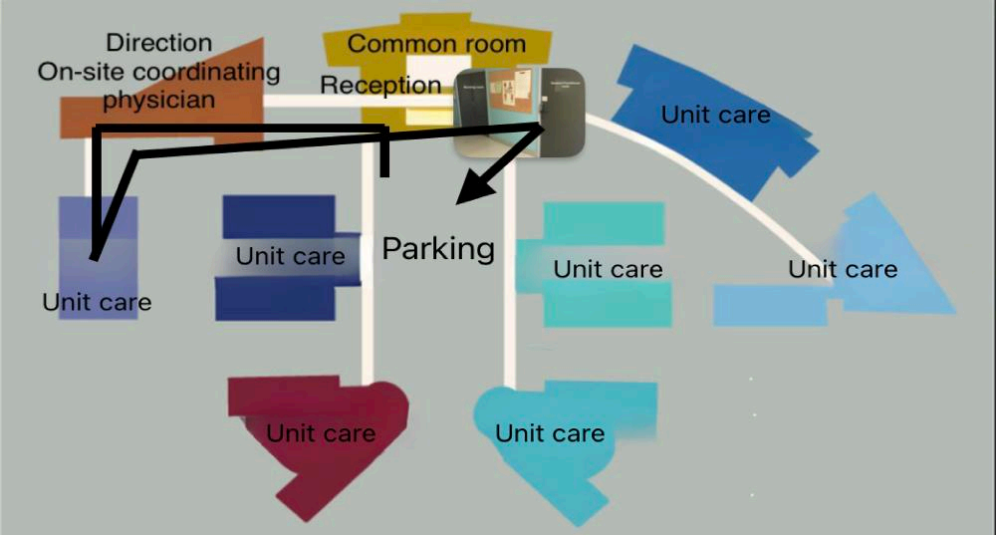
Pathway of a GP’s visit to NH 1. GP, general practitioner.

Second, the NH layout affected the opportunities for interprofessional collaboration between NH nurses and external private GPs visiting residents. Meeting seemed easier when GPs could write their prescription in the nursing room and when the nursing room was located on the path of the GP during the visit. In NH 3, for example, the nurse took the patient to a dedicated GP office connected to the nursing room (see Figure 2). In contrast, in NH 1, GPs could visit residents without any interaction with a nurse or an AN since the visit occurred directly in the resident’s room. Moreover, GPs wrote their prescription in a dedicated office disconnected from the nursing room and then put their prescription in a box in their office (see Figure 3).

### AP: a solely medical decision

AP in NHs appeared to be mostly an individual medical decision that did not involve any input from NH health professionals. The residents’ private GP or the concerned on-call private GP did not even discuss the details of their prescription with the NH nurses, who in turn felt unconsidered.

Observations in the afternoon of Day 3, NH 1

Around 5 pm, a resident’s private GP comes to visit them for a cough with no fever. This GP prescribed an antibiotic without any verbal contact with the nurse, who is busy preparing the residents’ treatments for the evening. He informs me that he is prescribing an antibiotic to treat rhinopharyngitis because he is afraid of complications due to the patient’s age and health condition. When the GP leaves, the nurse calls me and uses this example to criticize the lack of collaboration between GPs and nurses. She considers the GP’s behaviour as a sign of disdain and a source of misunderstanding: ‘I don’t understand why one resident needs such an antibiotic and others have no antibiotics. For example, Mrs. G has a long-term antibiotic prescription for lung problems, and the antibiotic does not change anything but still we use it. Another patient has been coughing for several weeks, and he got no antibiotics; it’s crazy!’

### Phone prescription as the most collaborative situation

To limit their workload and meet their time constraints, nurses perceived phone prescription as an effective alternative to onsite visits from residents’ GPs.

Moreover, they perceived this type of prescription, which was based on their description of the patient’s symptoms, as a sign of the GP’s trust in their clinical skills.

Observations at the end of the morning of Day 4, NH 3

The nurse calls the GP with the results of a resident’s urine culture. I listen to the exchange between the nurse and the resident’s private GP (without hearing the GP’s answers). The nurse communicates information about the resident and the results of the urine culture. She informs the GP that there is no need for him to come and visit the resident and that he will be able to regularize the antibiotic prescription during his next visit. After this exchange, the nurse expresses her satisfaction with working with GPs who trust her: ‘He gives me the name of the antibiotic by phone, and I note it in the file. This saves him a visit, and we get a quick answer. This general practitioner is easy to contact, he has many residents in our NH, and it is simple to work with him’.

## Discussion

Our investigation showed that interprofessional collaboration in the process of diagnosis and treatment of infections in NHs was critical through four steps: trigger, internal decision-making, call on external private GP and external GP intervention.

### Trigger

By triggering the process, ANs played a key role and were the actual gatekeepers in our study. Previous studies have shown the pivotal role of nurses in ordering urine cultures and triggering APs, as they provide a first-line assessment of residents and perceive early changes in their health status.^24^ As such, nurses have been reported as ‘gatekeepers’^25^ especially in AMS.^26^ The organization of care in French NHs relies on a nursing team inside NHs who often have poor access to resident’s external private GPs due to understaffing, which might partially explain the task shifting observed in our study. This finding calls for specific training targeting ANs to improve antibiotic use in NHs.

In our study, some nurses reported a lack of trust in ANs, which could hinder proper interprofessional collaboration regarding the diagnosis and treatment of infections at the first step of the process. This lack of trust was attributed to high AN staff turnover in NHs. AN staff turnover is associated with job demands (such as workload, emotional demands, work pace, conflicts and illegitimate tasks) and job resources (such as workflow, predictability, recognition, quality of leadership, vertical trust and interprofessional collaboration).^27^ Strömgren *et al.*^28^ reported that horizontal and vertical trust, in addition to social reciprocity and recognition, are associated with engagement in clinical improvement and quality of care.

Organizational clarity and improved vertical trust in NHs might thus be key to lowering staff turnover and implementing proper AMS programmes in such contexts.^29,30^

### Internal decision-making

In our study, internal decision-making regarding the diagnosis and treatment of infections in NHs involved various degrees of interprofessional collaboration—namely, full, incomplete or minimalist collaboration—and resulted in five types of action: performing microbiological tests, monitoring resident health status, calling on external private GPs, relying on internal coordinating GPs or transferring the resident to the hospital.

Interprofessional collaboration is needed to address complex problems such as the diagnosis and treatment of infections. This involves negotiations and interactions between professionals and includes 3D, i.e. partnership, cooperation and coordination.^31^

Interprofessional collaboration is defined as the process by which different health professionals work together to positively impact care.^32^ Accordingly, incomplete or minimalist interprofessional collaboration might lead to inappropriate practices, such as performing systematic urine dipstick testing in asymptomatic residents, leading to inappropriate use of antibiotics and affecting resident care overall. Promoting interprofessional collaboration between GPs and NH nurses should be considered in developing AMS suited to NHs, especially when critical stakeholders of AP and delivery (i.e. GPs, pharmacists and microbiologists) are external to these facilities. Interventions to promote interprofessional collaboration to improve professional practice and health outcomes have been systematically reviewed twice over the last decades and led to inconclusive results regarding their effectiveness.^33,34^ Which type of intervention to favour to promote interprofessional collaboration remains unknown, and these reviews highlighted the need for further evidence based on rigorous mixed-methods studies. The interventions reviewed included externally facilitated interprofessional activities, interprofessional rounds, interprofessional meetings and interprofessional checklists.^33^ Monthly interprofessional team meetings showed promising results on the reduction of psychotropic drug prescription in NHs.^35^ However, none of the included studies in these reviews focused on AMS. In a recent mapping of AMS tools for NHs, Jacquet *et al.*^36^ found no tools dedicated to interprofessional collaboration, but also tracking and reporting (audit and feedback). Collecting more information through sound observational research on infection prevention practices, nursing protocols and communication and actual monitoring of antibiotic use might also support AMS strategies in NHs.

In NHs, healthcare professionals often face complex care demands and turnover of nursing staff. These challenges might call for an interprofessional learning culture. A scoping review led by Verbeek *et al.* in 2023^37^ identified eight categories of facilitators of interprofessional learning culture in NHs: (i) shared language, (ii) shared goals, (iii) clear tasks and responsibilities, (iv) learning and sharing knowledge, (v) work approaches, (vi) facilitating and supporting change and creativity by the frontline manager, (vii) an open attitude and (viii) a safe, respectful and transparent environment. These facilitators could be used as levers to develop AMS interventions that promote an interprofessional learning culture.

### Call on external private GP and external private GP intervention

More challenging than internal interprofessional collaboration, NHs require interprofessional collaboration with external stakeholders directly contributing to residents’ care. Indeed, in French NHs, APs are mainly delivered by external private GPs (either resident’s GPs or on-call GPs).

In our study, interprofessional collaboration between NH health professionals and external GPs seemed to be facilitated in the case of remote prescribing, as compared with onsite prescribing while visiting a resident. Remote prescribing required external GPs to base their decision on clinical information accessible only through nurses’ reports. Remote prescribing might thus be seen as an opportunity to promote interprofessional collaboration. In addition, interprofessional collaboration has a goal: good patient care. Remote contact might thus also be advisable not only as a means to support GPs in deciding to prescribe but also in deciding to perform an onsite visit to the resident or not.

In our study, external GPs also provided onsite visits to residents with little to no contact with NH nurses, depending on the NH layout, indicating the critical value of environmental settings in any AMS intervention.

In both cases, i.e. remote prescribing and onsite GP visits, nurses and ANs should be considered as important an audience as GPs for AMS programmes in NHs. For instance, in addition to decision-making tools targeting GPs focusing on AP, such programmes should also include decision-making tools targeting nurses and ANs focusing on how and when testing urines, sputum and wounds, to further hinder potential inappropriate AP earlier in its process. In the same vein, Ramly *et al*.^16^ suggested that AMS programmes in NHs should include structured information tools, education of nurses and prescribers and organizational improvement.

In addition, we observed that the greater the number of external private GPs involved in resident care, the poorer the degree of interprofessional collaboration with NH nurses was. In a recent systematic review, Bocquier *et al.*^13^ also identified that a greater number of GPs involved in resident care was associated with higher volumes of AP. These results might argue in favour of limiting the number of GPs for resident care in each NH to further improve interprofessional collaboration and lower antibiotic use.

Our findings could be used to develop innovative AMS programmes that better fit NH settings. However, our study has several limitations: (i) our observations spanned a limited time frame, which might have prevented us from further investigating other critical aspects of AP, especially how the resident’s point of view was considered; (ii) one might criticize the lack of theoretical models underpinning our investigations. However, models investigating both individual (e.g. trust, attitudes, beliefs and knowledge) and environmental (e.g. social organizations and layout) attributes are still lacking in the literature, and an existing model might have prevented us from observing some important results derived from both types of attributes; (iii) due to a restrictive French regulatory context, we were not able to provide specific indicators of antibiotic consumption and prescriptions as DDJ/1000 bed-days for the three NHs in a reasonable publication timeframe. However, from the data collected in our study, these NHs did not present any major difference with the average French NHs.; and (iv) as applicable in qualitative studies, sample sizes in case studies are typically small.^38^ Nevertheless, if the few cases are carefully selected, literature recommends replicating the findings after reaching saturation in at least one supplementary case.^39^ In our study, due to limited human resources, we were not able to investigate a supplementary NH to fully meet this criterion.

## Supplementary Material

dlae205_Supplementary_Data
