# Supplementary material for: Exploration of interprofessional collaboration for the diagnosis of infections and antibiotic prescription in nursing homes using multiple case study observational research
Source: JAC Antimicrob Resist. 2025 Jan 13;7(1):dlae205. doi: 10.1093/jacamr/dlae205 (PMC11725391; doi:10.1093/jacamr/dlae205)
Supplement: dlae205_Supplementary_Data [file dlae205_supplementary_data.docx]

**Supplementary file 1**

**Table S1. Illustrative observation data on infection-related care in a nursing home**

| ***Overall understanding of the nursing home organization*** | | | |
| --- | --- | --- | --- |
| Context | **Nursing Home 1**  The first day is dedicated to the visit of the whole NH and its spatial organization. The observation takes place in the presence of the medical coordinator. | **Nursing Home 2**  The visit of the NH is proposed by the nurse coordinator during the first day of observation. I visit the nursing room, meeting room and medical office, administration. The nurse coordinator presents the spatial organization of the joint work of nurses, AN and the rest of the staff. | **Nursing Home 3**  On the first day, the NH director introduces me to the NH team and shows me the organization of NH: nursing room, meeting room and medical office, administration |
| Key field notes | Location: Entire NH  Residents are divided into care units. There are 7 care units. Each unit works in conjunction with another nearby unit, in lot of 2 units. Each lot is far apart, requiring nurses to use scooters to move from one lot to the next. In each lot, 2 ANs look after the residents. For all units, nursing care is provided by a single nurse. | Location: Entire NH  Residents' bedrooms are located on the first and second floors. The work organization of the nursing team, including nurses and AN, takes place between the nursing room and meeting room. The meeting room is a place to organize meetings between nurses, AN and coordinator staff (medical and nurse) and administration. The meeting room is a place for GPs. The GP can use a computer to track the observation. | Location: Entire NH  NH3 is a private, for-profit NH located in a village. NH has 24 beds, but an occupancy rate of 70%. The team consists of a nurse and 3 ANs every day. The problem described by the director is the difficulty of stability among his nurses and AN team. There is high staff turnover in this NH, with the need for the director to make up for staff shortages: nurse by training, he decides to replace himself nurses and even care assistants who are missing and not replaced, to ensure that the residents' care can be carried out. |
| ***Interprofessional exchanges within NH*** | | | |
| Context | **Nursing Home 1**  During days 2 & 3, observation is oriented toward the understanding of how the nurses and AN work together and how they organize their exchanges. | **Nursing Home 2**  Each observation day is oriented toward the understanding of how the nurses and AN work together and how they organize their exchanges. | **Nursing Home 3**  The second day of observations is oriented toward the understanding of how the nurses and AN work together and how they organize their exchanges. |
| Key field notes | Location: Nursing room, AN room  The organization of the nurses' and AN’s work is divided into half-days, with a morning team and an afternoon team. The 2 teams are required to exchange information on residents’ health status. A particularity of NH1 is that exchanges take place separately between the nurses and ANs. Information handovers take place at different times of the day and in different places. The only time for exchange between nurses and ANs is the monthly summary meeting with the medical and nurse coordinators. | Location: Nursing room, Meeting room  Time is set aside for exchanges at shift changeover times (night shift-morning shift, morning shift-afternoon shift). These exchanges take place with the presence of the entire nursing and AN’s team. At the start of the afternoon, there is a time for exchanges with the entire care team, as well as with the maintenance, coordinator staff and administration teams. These times enable a collective exchange of information on the residents and decisions to be taken to improve care. | Location: Meeting room  Nurses and ANs work 12-hour days. Every day, in the early afternoon, the duty nurse leads an exchange about the residents' problems. Positioned behind a desk, the nurse presents the residents one by one, their problems and the decisions taken (i.e., calling the general practitioner, organizing appointments with specialists, adapting follow-up, etc.). The nurse is the conductor of this time of exchange, with ANs playing a passive role. |
| ***Management of infectious diseases*** | | | |
| Context | **Nursing Home 1**  The second and third days is also dedicated to the observation of the process of management of infectious disease in NH | **Nursing Home 2**  The second and third day is dedicated to the observation of the process of management of infectious disease in NH. | **Nursing Home 3**  During each day of observation, time is dedicated to the observation of the process of management of infectious disease in NH. |
| Key field notes | Location: Care unit, Nursing room  I am in the nursing room in the late morning when the AN informs the nurse that a resident is presenting with a behavioral disorder and foul-smelling urine. The nurse asks the AN to obtain a urinary culture and then decides to call the resident's GP to organize a visit. After 7 minutes on the phone, the GP’s secretary informs her that she is leaving a message for the doctor and that he will see when he can come over. The doctor does not come during my observation day. The nurse waits for the results of the urinary culture before calling him back. | Location: Care unit, Meeting room  On my second day of observation, the nurse is alerted by an AN of a problem of unusual fatigue and cough in an 86-year-old resident. It is 3pm and, after taking vital parameters, the nurse decides to call the emergency GP: *“I know that this GP will not be able to visit the resident during the day”*. The nurse prefers to call the emergency GP, and their protocol stipulates that this care service should be called in case of urgent need and in the absence of the GP. In addition, the emergency GP is available and responds to requests much more easily according to the nurses than the GPs. | Location: Nursing room  On my last day of observation at the NH3, I notice a document from the emergency department on the desk in the nursing room. I ask the nurse about the reasons for this hospitalization: *“A resident developed a fever in the early evening, with an altered general condition and vomiting. There were no nurses at night, only AN. Faced with this problem and given that there was no emergency GP in the area, ANs called the emergency phone, which requested referral to the local hospital emergency department”* |
| ***Organization of exchanges between GP and nurse*** | | | |
| Context | **Nursing Home 1**  Days 2 & 3 enable the observation of the way exchanges between nurses and GPs are organized. | **Nursing Home 2**  The second day and third enable the observation of the way exchanges between nurses and GPs are organized. | **Nursing Home 3**  Days 3 & 4 are dedicated to the observation of the way exchanges between nurses and GPs are organized. |
| Key field notes | Location: Nursing room, GP’s room  GPs have a room at their disposal, which is located next to the nursing room. However, there seems to be no communication between them as I have observed: a GP arrives late in the morning to visit a resident with a nonfebrile cough. He parks his car and heads straight for the resident's care unit. After examining the resident, he writes prescriptions in a GP’s room, without passing through the nursing room. He places his prescription in a dedicated place and then leaves. The prescription is then picked up in the early afternoon by the nurse, who recorded it in the medical file. No exchanges between GP and nurse are observed. | Location: Meeting room  One early afternoon in NH, I observe the absence of exchanges between the GP and the nurse. A GP visits his resident following a positive urinary culture. After examining the patient in his room, he prescribes an antibiotic (fluoroquinolone) on the desk in the meeting room. The nurse works in the nursing room next to the meeting room, but no verbal exchange is observed, and no written record is made in the resident's file. | Location: Nursing room  During a visit by the only GP in the NH (another GP is working in the NH but is on maternity leave for several months and has not been replaced), the nurse brings a resident with advanced Alzheimer's dementia into the nursing room for alteration in general condition with increased confusion. The nurse presents the resident's problems. The exchange is cordial: *“it's good to have a sympathetic GP who listens. He knows his residents very well”* says the nurse. The clinical examination takes place in the nursing room in the presence of the nurse. The doctor asks the nurse to take a blood and urine culture and to monitor the patient's temperature and behavior. |

Abbreviations: ANs: assistant nurses; GPs: general practitioners; NHs: nursing homes
